# Combined Effect of Size and Charge on the Interaction of Nanoparticles with Mucus-Mimicking Mucin Hydrogels

**DOI:** 10.3390/ph18101498

**Published:** 2025-10-05

**Authors:** Natalia N. Porfiryeva, Ivan Zlotver, Alejandro Sosnik

**Affiliations:** Laboratory of Pharmaceutical Nanomaterials Science, Department of Materials Science and Engineering, Technion-Israel Institute of Technology, Haifa 3200003, Israel

**Keywords:** mucosal tissues, nano-drug delivery systems, mucus-mimicking mucin hydrogels, nanoparticle mucus diffusion

## Abstract

**Background/Objectives:** Understanding the interactions between nanoparticles and mucosal tissues is crucial for the development of advanced drug delivery systems, as the diffusion behavior of nanoparticles through mucus is strongly influenced by their size and surface properties, and the viscoelastic nature of the hydrogel matrix. In this study, we investigated the impact of nanoparticle size, surface charge, and hydrogel crosslinking density on nanoparticle diffusion in a mucus model in vitro. **Method:** Citrate-stabilized and PEGylated 30 and 100 nm gold nanoparticles were used as a model of nanoparticle and their diffusion through mucus-mimicking mucin-based hydrogels of two different crosslinking densities was assessed. **Results**: Citrate-stabilized 30 nm nanoparticles demonstrated greater diffusion in hydrogels mimicking native mucus compared to more densely crosslinked ones, reaching approximately 50.3 ± 0.2% diffusion within the first 5 min of the assay. This size-dependent effect was not observed for the 100 nm citrate-stabilized nanoparticles, which showed limited diffusion in both hydrogel types. To confer different surface charge, gold nanoparticles were functionalized by the conjugation of poly(ethylene glycol) (PEG) derivatives of identical molecular weight with different terminal moieties (neutral, and positively and negatively charged) to modulate the surface charge and assess their interaction with the negatively charged mucin matrix. PEGylated particles exhibited significantly greater mobility than their citrate-stabilized counterparts, regardless of size or hydrogel density owing to the muco-penetration effect of PEG. Among PEGylated particles, the neutral and negatively charged 30 nm variants demonstrated higher diffusion than the positively charged ones due to weaker interactions with the negatively charged mucin hydrogel. For the 100 nm particles, the neutral PEGylated nanoparticles exhibited greater diffusion than their positively charged counterparts. **Conclusions**: Overall findings could provide valuable insights into the more rational design of nanoparticle-based drug delivery systems targeting mucosal tissues.

## 1. Introduction

Moist surfaces of the body are covered by mucosal membranes, which play a crucial role as mediators of the interactions between the body and the external environment [1,2], and are ubiquitously distributed in different body sites, including the gastrointestinal tract, and the reproductive, ocular, respiratory, and excretory systems [3,4,5]. The function of mucosae is supported by a dense layer of mucus, a biological hydrogel with multiple essential roles [6,7,8]. Specifically, the mucus layer fulfills protective functions, preventing tissue dehydration, and the deleterious effect of mechanical, chemical, and biological insults and supporting the balance of the microflora, which is integral for its proper physiological performance [9,10]. It also hosts the so-called mucosa-associated lymphoid tissue, which is the most extensive component of the human lymphoid system [11]. The effectiveness of mucus as a primary protective barrier arises from its viscoelastic and adhesive properties that enable it to selectively control the passage of water, hormones, proteins, nutrients, and other essential molecules while simultaneously trapping harmful agents such as pathogens (e.g., bacteria and viruses) and exogenous particulate matter [12,13,14]. This functionality is largely attributed to its composition. Mucus contains ~90% water, which provides hydration and fluidity, while the remaining components consist of intertwined and cross-linked mucin fibers, a glycoprotein secreted by submucosal glands and goblet cells [15,16]. In addition to mucin, this complex mixture contains bacteria, cells, mineral salts, lipids, macromolecules, proteins, and cellular debris, all contributing to its multifaceted protective and functional roles [17,18,19]. The distinctive properties of mucus have garnered significant attention from researchers, establishing it as a promising platform for localized drug delivery and advancing the development of mucoadhesive and muco-penetrating drug delivery systems at the nano- and microscale size scales [20,21,22,23,24,25,26,27,28,29,30]. To enhance mucoadhesive or muco-penetrating properties, particles are often engineered by tailoring size, surface chemistry and charge, and shape, with a focus on the hierarchical, porous, and negatively charged structure of native mucus [24,31,32,33]. The mesh size delimited by mucin fibers has been estimated in the ~20–200 nm range, though particles with a diameter as large as 500 nm can still diffuse through the mucus pores when adhesive interactions are very weak [26,34] or by deformation upon the application of mechanical stress [35]. Following this rationale, polymers with a plethora of chemical structures, compositions, and charges have been investigated to synthesize nanoparticles for mucosal drug delivery [36]. Nevertheless, the molecular and structural features that control the interplay between nanoparticulate matter and mucus are not fully understood [37] and the prediction of these interactions based on the six theories of mucoadhesion (wetting theory, the electronic theory, the adsorption theory, the diffusion theory, the mechanical theory, and the fracture theory) remains challenging [38]. In previous research we showed that pure indinavir-free-based nanocrystals increase the oral bioavailability (estimated by the area-under-the curve) and the apparent half-life of this poorly water-soluble drug with respect to the free unprocessed counterpart by 21.9- and 27.5-fold, respectively, in mongrel dogs [39]. A possible explanation for these unexpected results is that upon oral administration the poorly water-soluble nanocrystals undergo entrapment within the intestinal mucus layer, and slow dissolution and intestinal absorption over time. A better understanding of the fundamental features that govern particle–mucus interactions and their diffusion-driven penetration is crucial to rationally design mucosal drug delivery systems and predict their performance in vivo. We proposed a theoretical model that describes the local interactions between particles and biological gels, and the particle penetration phenomenon under both spontaneous and forced conditions [40]. This model did not consider the influence of particle size, concentration, or changes in gel porosity on these interactions. More recently, an experimental–theoretical approach has been proposed to predict the effect of particle size and concentration, and hydrogel crosslinking density, on diffusion-driven particle penetration in vitro [41]. For this, porcine gastric mucin type II-based hydrogels that mimic the rheological (storage and loss modulus) and structural properties (e.g., porosity) of native intestinal mucus were synthesized by methacrylation and photo-initiated free radical crosslinking, and the effect of particle size and concentration on the interaction of pure nanoparticles of the tyrosine kinase inhibitor dasatinib characterized. The model was challenged in a preliminary oral pharmacokinetics study in rats that showed a very good correlation with the in vitro results [41]. In this model, the particle surface charge, a key property affecting the interaction with the negatively charged mucus, was not considered. A major drawback in this work was the limited reproducibility and cumbersome synthesis of mucin hydrogels. To overcome this issue, we developed a simple, efficient and reproducible synthetic pathway of mucus-mimicking mucin-based hydrogels by a tandem chemical and physical crosslinking [42].

A critical aspect in studying nanoparticle–mucus interactions is the ability to use particles with tunable surface charge without resorting to complex synthesis. In this regard, gold nanoparticles offer an advantage, as their surface charge can be readily modified through simple functionalization with PEG blocks bearing terminal moieties with variable charge. They have also already been successfully employed to study particle penetration through the skin [43]. In addition, their optical properties enable sensitive and quantitative detection on hydrogel surfaces, making them a promising model system for probing size- and charge-dependent diffusion in mucus-mimicking hydrogels.

Aiming to gain an insight into the combined effects of size and surface charge on the interaction of nanoparticles with mucosal tissues, in the present work, we synthesize 30 and 100 nm gold nanoparticles [44] surface-modified with poly(ethylene glycol) (PEG) derivatives displaying neutral, positive, and negative terminal moieties [45], and their penetration behavior in mucin-based hydrogels of two different cross-linking densities, and consequently porosity, assessed. Overall findings give an additional insight into the interaction between nanoparticles and mucus and could contribute to further understanding the key features of particulate matter towards a more rational design and application.

## 2. Results

In our previous work [42], we synthesized mucus-mimicking mucin-based hydrogels by a tandem chemical (glutaraldehyde) and physical (one or two freeze–thawing cycles) crosslinking method of mucin solutions. As reported, hydrogels produced with one freeze–thawing cycle show water content of 97.6–98.1%, density of 0.0529–0.0648 g cm^−3^, and storage and loss moduli of ≈40–60 and ≈3–5 Pa, respectively, along with a highly porous structure featuring a mixture of large and small pores, closely resembling the properties of native gastrointestinal mucus [42]. After two freeze–thawing cycles, mucin hydrogels show mechanical properties that might resemble pathological mucus.

### 2.1. Synthesis and Characterization of Gold Nanoparticles

To investigate particle interactions with mucus, we used mucin-based hydrogels crosslinked with 0.75% *w*/*v* GA and subjected to one or two freeze–thawing cycles (named in this work 1-Cycle and 2-Cycle, respectively) that result in viscoelastic properties mimicking natural gastrointestinal mucus (1-Cycle) or sturdier ones suitable in tissue engineering and biosensing applications (2-Cycle). These hydrogels were applied to systematically assess how particles of the two sizes and surface charges interact with these hydrogels, while also examining the effect of crosslinking density. For this study, we initially synthesized gold nanoparticles with two size ranges, with Z-average diameters of 26 ± 6 nm (referred in this work as 30 nm nanoparticles) and 96 ± 5 nm (referred here as 100 nm nanoparticles), as measured by dynamic light scattering (DLS), and surface-stabilized with citrate ions (Table 1).

**Figure 1:**
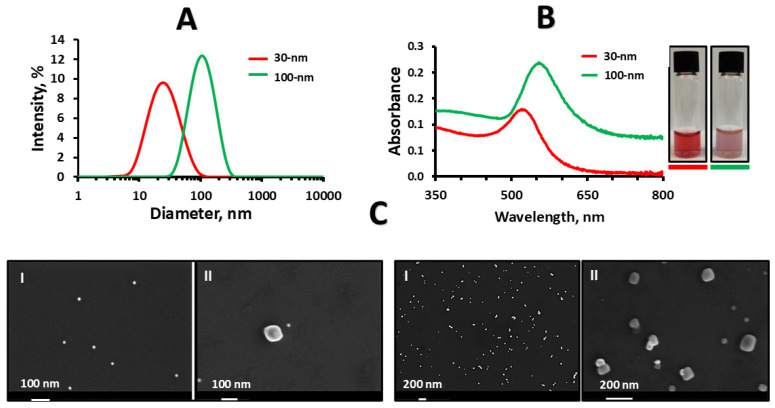
Characterization of sodium citrate-stabilized gold 30 and 100 nm gold nanoparticles. (**A**) Size distribution by intensity, as determined by DLS at 25 °C. (**B**) UV–Vis spectra of the nanoparticles. Images show the characteristic colors of these nanoparticles after stabilization by citrate ions. (**C**) HR-SEM micrographs of (**I**) 30 nm and (**II**) 100 nm nanoparticles, nano-sprayed onto the surface of a silicon wafer at two different magnifications.

Nanoparticles showed a monomodal size distribution (one size population, Figure 1A) and small polydispersity index (PDI) values of 0.15 ± 0.05 and 0.21 ± 0.01, respectively, indicating the presence of a narrow size range and a uniform particle population (Table 1). Sodium citrate was used in the synthesis as a reducing agent and stabilizer and imparted a negative charge to the nanoparticle surface [44], with Z-potential values of −17 ± 3 and −17 ± 1 mV for 30 and 100 nm nanoparticles, respectively (Table 1). We also characterized the 100 nm nanoparticle suspension by nanoparticle tracking analysis (NTA); the Brownian motion is exemplified in Supplementary Video S1. A size of 95 ± 3 nm and a concentration of 1.87 × 10^9^ ± 4.17 × 10^8^ particles/mL (after correction by the dilution factor) was measured (Figure S1). UV–visible spectroscopy confirmed the presence of localized surface plasmon resonance exhibited by the nanoparticles, as indicated by strong bands in the visible region with λ_max_ at 524 nm for 30 nm and 570 nm for 100 nm nanoparticles (Figure 1B). The typical rounded morphology and smooth surface of gold nanoparticles stabilized by citrate ions was confirmed by high resolution scanning electron microscopy (HR-SEM analysis), as shown in Figure 1C; these results agreed with [46]. The nanoparticle size measured by HR-SEM was in very good agreement with DLS and NTA results.

To investigate the combined effect of nanoparticle size and surface charge on their interaction and diffusion through mucus-mimicking mucin-based hydrogels, nanoparticles were coated by the conjugation of PEG derivatives of identical molecular weight of 2000 g/mol, though displaying different terminal moieties: (i) a terminal -OCH_3_ moiety and neutrally charged (named PEG), (ii) a terminal primary amine moiety and positively charged (named PEG-NH) and a terminal carboxyl moiety and negatively charged (named PEG-CM). Different charge densities are anticipated to affect the electrostatic interaction of the nanoparticles with negatively charged mucin molecules in the hydrogel. The size and the surface charge (estimated by the Z-potential) of the PEGylated nanoparticles are summarized in Table 1. As expected, PEGylation led to a slight increase in the size of the nanoparticles owing to the presence of a hydrated PEG corona on their surface [47]. The use of different types of thiolated PEG ligands resulted in the anticipated changes in the Z-potential (an estimation of the surface charge) of 30 nm nanoparticles from −18 ± 3 for non-PEGylated to −3 ± 1, −20 ± 2, and +9 ± 1 mV for PEG-, PEG-CM-, and PEG-NH-modified ones, respectively. Similar changes were observed for the 100 nm nanoparticles, Z-potential values shifting from −17 ± 1 mV for the citrate-stabilized ones to −4 ± 1, −17 ± 1, and + 17 ± 1 mV, for PEG-, PEG-CM-, and PEG-NH-modified counterparts, respectively. Overall, this flexible synthetic strategy enables the synthesis of colloidally stable nanoparticles with different sizes and surface charge properties.

### 2.2. Particle Diffusion into Mucus-Mimicking Mucin-Based Hydrogels In Vitro

#### 2.2.1. Effect of Nanoparticle Size

**Figure 2:**
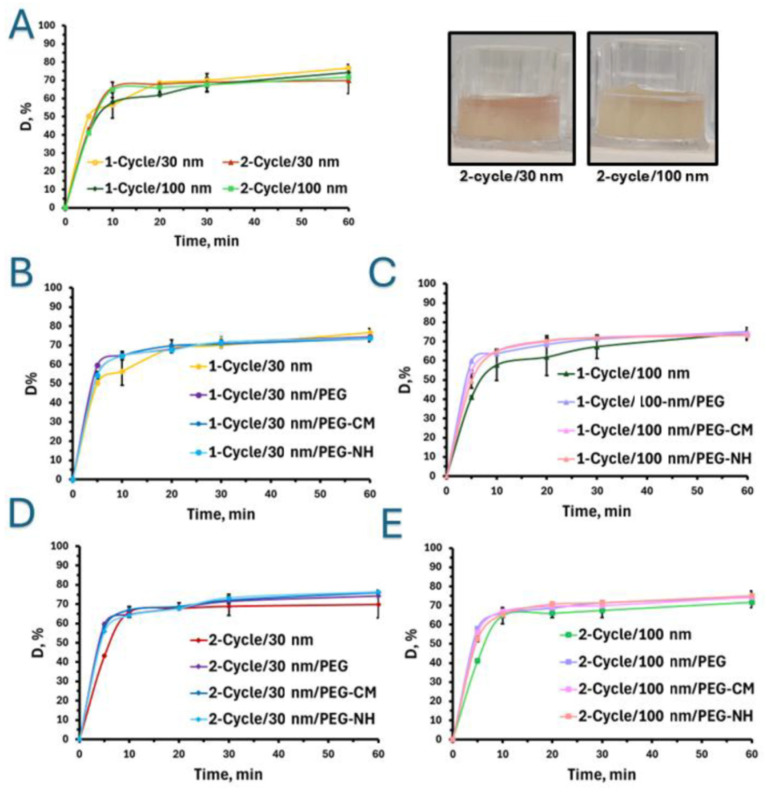
Diffusion (D, expressed in % of the initial nanoparticle concentration) of citrate-stabilized gold nanoparticles through mucin-based hydrogels prepared using one (1-Cycle) and two (2-Cycle) freeze–thawing cycles. (**A**) Comparison of 30 and 100 nm nanoparticle diffusion through mucin hydrogels after 1-Cycle and 2-Cycle treatments. (**B**) Diffusion of 30 nm non-PEGylated and PEGylated nanoparticles through (**B**) 1-Cycle and (**C**) 2-Cycle mucin hydrogels. (**D**) Diffusion of 100 nm non-PEGylated and PEGylated nanoparticles through (**D**) 1-Cycle and (**E**) 2-Cycle mucin hydrogels. Images in A: Hydrogels after 2-cycle showing diffusion of 30 and 100 nm non-PEGylated nanoparticles after 5 min. Results expressed as Mean ± S.D. (*n* = 3). A *p*-value of <0.05 was considered statistically significant.

Following the comprehensive characterization of the nanoparticles, mucin hydrogels were utilized to evaluate diffusion-driven particle diffusion. Freshly prepared citrate-stabilized 30 and 100 nm nanoparticles [44] were evenly distributed on the surface of mucin-based hydrogels with two crosslinking densities (and rheological properties) and the diffusion was assessed. The different setups are named 1-Cycle/30 nm, 2-Cycle/30 nm, 1-Cycle/100 nm and 2-Cycle/100 nm. Figure 2A summarizes the results of nanoparticle diffusion through the hydrogels at different timepoints over 1 h. Results indicated that, regardless of the nanoparticle size, ~75% of them successfully penetrated both hydrogels. Furthermore, after the first 5 min of the assay, a statistically significant difference in nanoparticle diffusion was observed only for the 1-Cycle/30 nm group, reaching 50.3 ± 0.2%, whereas diffusion levels for 2-Cycle/30 nm, 1-Cycle/100 nm, and 2-Cycle/100 nm were in the 41–43% range, differences among them being statistically insignificant. Thirty-nanometer nanoparticles penetrated the hydrogel after 1-Cycle, which mimics intestinal mucus, more rapidly than the 100 nm counterparts. After 10 min, no significant differences were observed between the two types of hydrogels for the 30 nm non-PEGylated nanoparticles sizes. At subsequent timepoints, no statistically significant differences were detected across the other samples.

**Figure 3:**
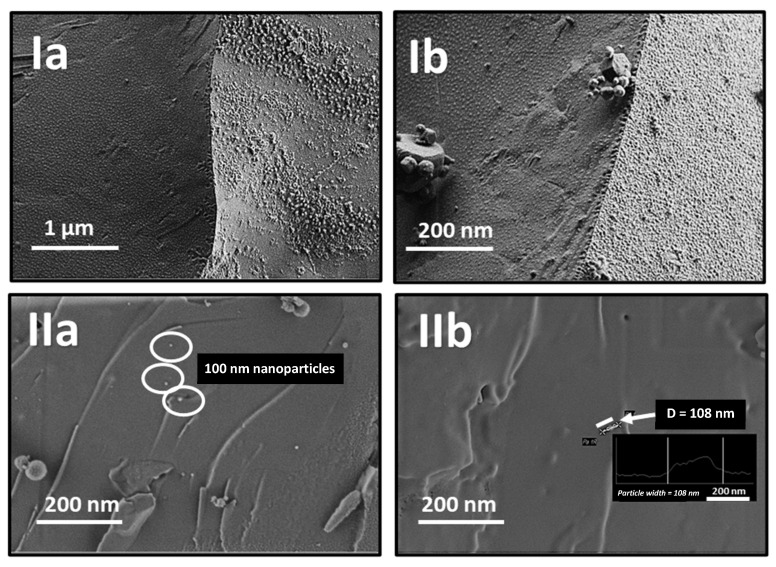
Cryo-SEM micrographs of a swollen mucus-mimicking mucin hydrogels after two freeze–thawing cycles, captured at an acceleration voltage of 0.8 kV: (**I**) the overall hydrogel structure, and (**II**) 100 nm nanoparticles observed within the hydrogel at different magnifications. (**Ia**) 20k, (**Ib**) 30k, (**IIa**) 40k, and (**IIb**) 50k. White ellipses in IIa point out some of the 100 nm gold nanoparticles visualized inside the mucin hydrogel matrix. The inset image in IIb shows the particle width profile of the nanoparticle pointed out with the white arrow with a diameter (D) of 108 nm, as measured in the microscope.

The ability of these nanoparticles to diffuse into the hydrogels was confirmed by cryogenic scanning electron microscopy (cryo-SEM) analysis, which captures the internal structure of the swollen hydrogels from the top and the side simultaneously, allowing clear visualization of its surface architecture (Figure 3Ia). The diffusion of 100 nm nanoparticles through the hydrogel is evident in a cross-section of the hydrogel, confirming their presence within the structure (Figure 3IIb), where a distinctively smooth inner surface characteristic of biological hydrogels, along with an intact hydrogel network structure, was also with an intact hydrogel network structure; these results were in good agreement with [41,48,49].

#### 2.2.2. Effect of Particle Charge and Hydrogel Crosslinking Density

In the present study, hydrogels were engineered to mimic both the viscoelastic characteristics of native intestinal mucus and the more densely crosslinked structure of pathological mucus by regulating the number of freeze–thawing cycles applied during preparation. The results revealed that the primary differences in the diffusion of particles with varying surface charges and sizes occurred within the first 5 min of the assay, regardless of their size and crosslinking density. After this initial period, no statistically significant changes in diffusion (D) were observed (Figure 2A–E). Notably, during the initial time frame of 5 min, the diffusion of all PEGylated nanoparticles was significantly greater than that of citrate-stabilized nanoparticles.

Specifically, after 5 min, neutral 30 nm nanoparticles (1-Cycle/30 nm/PEG) exhibited a D value of ~60%, compared to 54.8 ± 0.5% and 54.4 ± 1.9% for the negatively and positively charged PEGylated counterparts, respectively, and 50.3 ± 0.2% for negatively charged citrate-stabilized 30 nm nanoparticles (1-Cycle/30 nm) (Figure 2B). Negatively charged particles also demonstrate reduced diffusion due to electrostatic repulsion from the negatively charged mucin. This trend was also evident in the denser mucin hydrogels (Figure 2C), with diffusion rates after 5 min for neutral, negatively, and positively charged PEGylated nanoparticles with D values of 64.2 ± 0.9% (2-Cycle/30 nm/PEG), 57.7 ± 1.5% (2-Cycle/30 nm/PEG-CM), and 55.8 ± 0.4% (2-Cycle/30 nm/PEG-NH), respectively, while the D value for citrate-stabilized 30 nm nanoparticles was significantly lower, 43.2 ± 0.4% (Figure 2C). In the case of 100 nm nanoparticles in 1-Cycle hydrogels, a statistically significant difference in diffusion after 5 min was also observed due to variations in surface charge, with some distinct behaviors (Figure 2D). Neutral PEGylated nanoparticles showed statistically higher diffusion compared to their positively charged counterparts, with D values of 59.8 ± 0.5% (1-Cycle/100 nm/PEG) versus 49.6 ± 3.9% (1-Cycle/100 nm/PEG-NH_2_). Notably, the diffusion of citrate-stabilized nanoparticles was even lower than that of all PEGylated counterparts, with D values of 41.1 ± 0.6% (1-Cycle/100 nm). Hydrogels subjected to two-cycle freeze–thawing exhibited similar diffusion profiles (Figure 2E), with 100 nm particle diffusion after 5 min reaching D of 41.1 ± 0.6% for citrate-stabilized nanoparticles (2-Cycle/100 nm). A statistically significant difference among the PEGylated nanoparticles was observed between the neutral and positively charged particles, with D values of 57.9 ± 0.4% for neutral (2-Cycle/100 nm/PEG) and 52.5 ± 1.3% for positively charged nanoparticles (2-Cycle/100 nm/PEG-NH).

## 3. Discussion

Mucosal tissues represent a major interface between the body and the external environment and are covered by a highly hydrated mucins gel called mucus. Mucus lubricates, protects, and modulates the moisture levels of the tissue and is capitalized in transmucosal drug delivery. Pharmaceutical researchers often use freshly excised animal mucosal membranes to assess mucoadhesion and muco-penetration of pharmaceutical formulations, which may struggle with limited accessibility, reproducibility, and ethical questions. To study particle interactions with mucus, we utilized mucin-based hydrogels cross-linked with 0.75% *w*/*v* glutaraldehyde and subjected to either one or two freeze–thaw cycles (referred to in this work as 1-Cycle and 2-Cycle, respectively). These hydrogels exhibited viscoelastic properties comparable to natural gastrointestinal mucus or, in the case of the 2-Cycle formulation, enhanced robustness suitable for other biomedical applications. More densely crosslinked and viscous mucin hydrogels could also mimic the properties of pathological mucus such as in cystic fibrosis [50]. These hydrogel formulations were utilized to systematically evaluate the interactions between the hydrogel and particles of varying sizes and surface charges, as well as to investigate the influence of the crosslinking density. In this context, gold nanoparticles were selected as a model system due to their well-known optical and plasmonic properties that enable easy quantification by UV-Vis spectrophotometry, the size and shape can be controlled by the synthetic parameters, and the surface can be modified by the conjugation of thiolated PEG derivatives [51,52,53]. At the same time, experience has been accumulated in using gold nanoparticles to study particle penetration into mucus, providing insights into biomedical processes and particle behavior with mucins under different physiological conditions, particularly variations in pH [54,55]. Based on these considerations, we produced spherical gold nanoparticles because this is the most common shape of different polymeric and lipidic nanocarriers used in mucosal drug delivery. Synthesized nanoparticles, according to the literature, showed PDI values within the range considered monodisperse [54,55] and their negative zeta potential (Z-potential) values were consistent with the characteristic surface charge of citrate-stabilized gold nanoparticles [56]. The absorption pattern and maximum values (λ_max_) are characteristic of the surface plasmon resonance of gold nanoparticles, resulting from the interaction between the incident light and the conduction electrons [57]. These spectral absorption features and corresponding colors of synthesized nanoparticles are consistent with previous reports, confirming the presence of quasi-spherical nanoparticles of the desired size range [47,58]. At the same time, the use of thiolated PEG conjugation for nanoparticles serves a dual role, acting both as an additional physical stabilizer and as a steric barrier, thereby ensuring colloidal stability and preventing particle aggregation [59,60].

Following comprehensive nanoparticle characterization, mucin hydrogels were used to investigate diffusion-driven particle transport. A previous report demonstrated that 100 and 200 nm nanoparticles diffuse more efficiently in pulmonary mucus compared to larger particles (500 nm) [61], which is consistent with our results, showing the penetration of approximately 75% of the nanoparticles into the hydrogel within one hour for both particle sizes. As described above, the mesh size in native mucus is in the 20–200 nm range [26]. The results highlight how size differences, even within the mesh range of mucus, can affect diffusion. Smaller nanoparticles penetrated the less crosslinked hydrogel, which mimics intestinal mucus, more rapidly than the 100 nm particles. A similar effect was observed for small viruses up to 55 nm in diameter, which demonstrated the ability to diffuse through cervical mucus at rates comparable to those in water, whereas larger viruses (approximately 180 nm in diameter) were found to diffuse more slowly in mucus than in water [62]. The absence of a statistical difference at other times points likely stems from the relatively small size of the nanoparticles used in this study, which are smaller than the larger mesh size reported for mucus (~200 nm); our attempts to produce larger ones were not successful. In previous work investigating the effect of particle size and concentration, and mucin hydrogel crosslinking density, we observed significant differences in particle penetration into the hydrogels between 200 nm and 1.2 and 1.33 mm particles [41], with the most pronounced changes occurring during the first 5 min of the assay.

Surface charge is recognized as a key factor in determining interactions within biological hydrogels such as mucus, where negatively charged mucin exhibits selective affinity for particulate matter with varying surface charges [63,64]. To modulate this parameter, gold nanoparticles were surface functionalized with PEG blocks of identical molecular weight but differed in the terminal functional groups that impart the surface charges. While surface charge governs the nature and strength of particle–mucin interactions, crosslinking density represents another critical physicochemical factor that significantly impacts nanoparticle diffusion by directly affecting the rheological properties and the porosity of the hydrogel matrix [65]. This is particularly relevant given the substantial variability in the rheological properties of mucus across different regions of the human body and even within the same system (e.g., along the gastrointestinal tract) [13,36]. To address this, our tandem chemical–physical crosslinking method easily enables the control of the crosslinking density and the rheological properties in a wide range of mucin-based hydrogels [42].

As is known, the presence of a PEG corona forms a hydrated steric stabilization layer around the particles that prevents aggregation and reduces adhesive interactions with mucin [47]. The behavior of the PEGylated nanoparticles is also consistent with previously reported findings, which indicate that neutral particles exhibit enhanced diffusion due to minimal interactions with negatively charged mucin resulting from reduced hydrophobic and electrostatic interactions [66]. This phenomenon has been coined muco-penetration. Electrostatic interactions are primarily responsible for the observed differences: negatively charged particles experience repulsion from the negatively charged mucin glycoproteins, whereas neutral particles are predominantly impeded by steric constraints imposed by the entangled mucin network. Conversely, positively charged particles tend to more strongly interact with negatively charged mucin glycoproteins, which contributes to the highly adhesive nature of mucin. Our results showed pronounced diffusion of neutral particles compared to positively charged particles and are in line with previous studies, indicating that nanoparticles functionalized with primary amine groups exhibit limited diffusion in mucus, likely due to physical hindrance from the entangled mucin network or stronger adhesive interactions with mucin glycoproteins network [26]. Nonetheless, the presence of a PEG-corona continues to enhance the diffusion of PEGylated nanoparticles compared to citrate-stabilized ones. These findings underscore the critical role of crosslinking density, particle size, and surface charge in governing diffusion behavior through mucus-mimicking hydrogels.

Undoubtedly, for further understanding the processes occurring during the interaction of nanoparticles with mucus and increase the ability of this simple experimental setup to predict the interaction of nanoparticles with mucus under physiological conditions, studies incorporating flow are required. In this framework, more comprehensive studies could include ex vivo validation on porcine mucosal tissues, as well as in vivo investigations to establish correlations with pharmacokinetic profiles. Having pointed this out, the method proposed in this work represents one approach to understanding the processes occurring at the initial stage and provides an opportunity to assess the relevance of the experiments conducted.

## 4. Materials and Methods

### 4.1. Materials

Mucin (gastric porcine, type II) was supplied by Sigma–Aldrich (St. Louis, MO, USA). Glutaraldehyde (25% *w*/*v* solution in water) and hydrogen tetrachloroaurate (III) hydrate Premion^TM^ (HAuCl_4_, 99.995% metal basis, Au 49% min) were supplied by Thermo Fisher Scientific (Waltham, MA, USA). Sodium citrate dihydrate was purchased from Spectrum Chemicals Mfg. Corp. (New Brunswick, NJ, USA). Nitric acid (HNO_3,_ 70% *w*/*v*) and sodium hydroxide pearls were obtained from Bio-Lab Ltd. (Jerusalem, Israel). Hydrochloric acid (HCl, 37% *w*/*v*) was received from Daejung Chemicals & Metals Co. (Siheung-si, Republic of Korea). Heterobifunctional carboxymethyl-poly(ethylene glycol)-thiol (PEG-CM, molecular weight of 2000 g/mol), amine-poly(ethylene glycol)-thiol (PEG-NH, molecular weight of 2000 g/mol), and monofunctional methoxy-poly(ethylene glycol)-thiol (PEG, molecular weight of 2000 g/mol) were purchased from Laysan Bio Inc. (Arab, AL, USA). Dialysis tubing CelluSep T1 with a molecular weight cut-off of 3500 Da was received from Membrane Filtration Products Inc. (Seguin, TX, USA). Milli-Q water obtained from the Milli–Q^®^ Direct 8 Water Purification System (Merck Millipore, Molsheim, France) was used throughout the experiments involving aqueous solutions. All other chemicals were of analytical grade as supplied without modification.

### 4.2. Preparation of Mucus-Mimicking Mucin-Based Hydrogels

Mucus-mimicking mucin-based hydrogels were synthesized according to a previous report [42]. Briefly, a mucin solution in water was purified by dialysis against for three days, followed by centrifugation (10 min, 2500 rpm, 221.12 V01 rotor, Hermle Z300, Labortechnik GmbH, Wasserburg am Bodensee, Germany). The supernatant was frozen in liquid nitrogen and freeze-dried using a Labconco FreeZone 4.5 Plus (Kansas City, MO, USA). Next, 960 µL of a 4% *w*/*v* aqueous solution of dialyzed mucin was poured into 24-well plates, mixed with 37% HCl (10 µL) for 20 min, and incubated for 1 h with 25% *w*/*v* glutaraldehyde (30 µL). The well-plates were subjected to one or two freeze–thawing cycles (24 h of freezing at –20 °C, followed by 4 h of thawing at 25 °C), and washed multiple times overnight with Milli-Q water.

### 4.3. Preparation of Gold Nanoparticles

Gold nanoparticles with different sizes and stabilized with sodium citrate were synthesized using the Turkevich method [44,66,67,68]. Initially, all glassware and magnetic stirrers were cleaned using aqua regia in a 3:1 (*v*/*v*) ratio of HCl to HNO_3_, followed by thorough rinsing with Milli-Q water and drying overnight. Subsequently, a HAuCl_4_ solution in Milli-Q water (0.45 mM, 50 mL) was added to a 250 mL round-bottom flask and heated under reflux (100 °C). Once the solution boiled, a 1% *w*/*v* aqueous sodium citrate solution (5 mL) was quickly added under magnetic stirring (300 rpm). Within the first minute, the solution color changed from violet to a wine-red. The reaction mixture was stirred for 30 min, then allowed to cool to room temperature (RT) under constant stirring to prepare uniform nanoparticles, before being filtered using an Acrodisc^®^ syringe filter (0.2 µm, Pall Gelman, Port Washington, NY, USA). Using this procedure, gold nanoparticles with an approximate size of 30 nm were synthesized. To prepare 100 nm nanoparticles, a seed-mediated method was utilized [69]. For this, a 30 nm nanoparticle dispersion synthesized above was diluted in Milli-Q water (39.537 mL) over 5 min. Subsequently, a 44.7 mM HAuCl4 solution (227 μL) was added to the suspension and mixed for 1 min under magnetic stirring (300 rpm). Finally, a 1% *w*/*v* aqueous sodium citrate solution (176 μL) was poured into the reaction mixture and left under stirring overnight.

PEGylated 30 and 100 nm gold nanoparticles were prepared by reacting thiolated PEG derivatives (PEG, PEG-NH, or PEG-CM) with the citrate-stabilized nanoparticles according to a previous report with minor changes [70]. Briefly, PEG solutions in water with different concentrations (10 μL aliquots) were added to a nanoparticle suspension (1 mL) to achieve final PEG concentrations of 10, 1, 0.1, and 0.01 mM. The mixture was vortexed and incubated for 2 h, at RT. All the samples were stored at 4–8 °C until further use.

### 4.4. Characterization of the Nanoparticles

Nanoparticles were characterized by UV-Vis spectrophotometry using a Multiskan GO Microplate Spectrophotometer (Thermo Fisher Scientific Oy, Vantaa, Finland) in the wavelength range of 350–800 nm. Skanlt^TM^ Software for Microplate Readers (Thermo Fisher Scientific Oy) was utilized for recording. All the measurements were performed at 25 °C and Milli-Q water was used as a blank.

The size (expressed as hydrodynamic diameter, D*_h_*), PDI (an estimation of the size distribution), and zeta-potential (Z-potential, an estimation of the surface charge) of the different nanoparticles were measured using a Zetasizer Nano-ZS (Malvern Instruments, Malvern, UK) equipped with a 4 mW He–Ne laser (λ = 633 nm) at a scattering angle of 173°, an absorption coefficient of 1.520, and a refractive index of 0.100. Samples (1 mL) were poured into RED CUV010015 cuvettes (ALEX RED Ltd., Mevaseret Zion, Israel) and DTS−1070 folded capillary tube cuvettes (Malvern Instruments), for the determination of size and PDI, and Z-potential, respectively. Each sample was measured at least three times at 25 °C and the data were analyzed utilizing CONTIN algorithms (Malvern Instruments).

The nanoparticle size, concentration in suspension (expressed as particles per mL), and Brownian motion were characterized using NTA with a NanoSight NS500-Zeta HSB system equipped with a high-sensitivity SCMOS camera (Malvern Instruments), at 25 °C. For the analysis, the nanoparticle suspension was diluted (1:10) with Milli-Q water and loaded onto the NTA pump. Three 60 s videos were recorded for each of the three distinct samples. The tracking of the nanoparticles was captured and processed using NTA 3.2 software (Malvern Instruments).

The morphology of unmodified gold nanoparticles was visualized by HR-SEM utilizing a Zeiss Ultra-Plus Zeiss Ultra-Plus FEG-SEM (Carl Zeiss SMT GmbH, Oberkochen, Germany). For this, a nanoparticle dispersion (10 µL) was nano-sprayed twice onto the top of a silicon wafer (CZ polished silicon wafers <100> oriented, highly doped N/Arsenic, SEH Europe Ltd., Livingston, UK) using high-pressure nitrogen to facilitate the uniform distribution of the nanoparticles on the surface. Then, the wafer with the sample was dried overnight in the vacuum desiccator. The samples were visualized at 1–4 kV in the range of 3–5 mm working distance, and in an in-lens secondary electron condition.

### 4.5. In Vitro Particle Diffusion Studies on Mucus-Mimicking Hydrogels Top of Form

The diffusion of the different nanoparticles in mucus-mimicking mucin-based hydrogels was investigated using a slightly modified protocol [41]. For this, two mucin hydrogels of different crosslinking density and rheological properties were used, each subjected to either one or two freeze–thawing cycles and referred to as 1-Cycle and 2-Cycle, respectively. Hydrogels with a diameter of 2 cm and a thickness of 3 mm were prepared in 24-well plates. Gold nanoparticles with a diameter of ~30 nm and ~100 nm, as measured by DLS, before the PEGylation step and at a concentration of 71 µg/mL, which was the concentration used in our previous work [41], were used to investigate the diffusion properties through the hydrogels. Initially, 200 µL aliquots of the nanoparticle stock suspension were carefully placed at the center of the hydrogel surface and an additional 200 µL of water was added to form a 3.0 mm liquid column above it. Hydrogels were incubated with the nanoparticles for 5, 10, 20, 30, and 60 min, at RT. After each timepoint, the supernatant was collected, and the hydrogel surface was gently washed to retrieve all the nanoparticles that did not penetrate the hydrogel. The gold nanoparticle concentration in the supernatants was analyzed using UV–vis spectrophotometry at 524 and 670 nm, corresponding to the absorbance maxima of 30 and 100 nm nanoparticles, respectively. The concentration of the nanoparticles in the supernatant was quantified by interpolating the absorbance in calibration curves built using nanoparticle suspensions in the 0.05–1 mM concentration range (R^2^ = 0.9966 for 30 nm and R^2^ = 0.9995 for 100 nm nanoparticles). The percentage of nanoparticles that penetrated the hydrogels expressed as diffusion percentage, D (%), was determined according to Equation (1).

(1) $$D\;(\%)=\frac{C_{i}-C_{f}}{C_{f}}\times 100$$

where C_i_ and are the initial and final gold nanoparticle concentrations in the suspension before after the penetration test, respectively.

All studies were conducted in triplicate and expressed as Mean ± S.D. Hydrogels without nanoparticles and treated with an identical water column were used as a blank. Only one parameter (nanoparticle size, surface charge, or hydrogel crosslinking density) was modified every time, while keeping the remaining constant.

The structure of mucin-based hydrogels before and after diffusion studies was carried out using cryo-SEM. Samples were analyzed in a Zeiss Ultra-Plus High-Resolution Cryo-SEM (Carl Zeiss SMT GmbH) fitted with a Schottky field-emission electron gun and a Leica VT100 cryo-system (Leica Biosystems, Nussloch, Germany). In this procedure, the middle side of the sample was sliced using a scalpel and placed between two aluminum plates (d = 3 mm) with a gap of 100 µm. Subsequently, the specimen was vitrified in an EM ICE system (Leica Microsystems, Wetzlar, Germany) using a liquid-nitrogen jet at 2100 bar for 360 ms. Then, the morphology of specimens without coating was imaged at −150 °C at a low acceleration voltage.

### 4.6. Statistical Analysis

All measurements, unless otherwise noted, were carried out in triplicate. Statistical significance was analyzed using GraphPad Prism software (version 10.0). The statistical differences among more than two groups were compared and assessed for significance through a one-way analysis of variance (ANOVA), whereas for two-group comparisons, an independent samples *t*-test was employed for each pair of datasets. A *p*-value of <0.05 was considered statistically significant.

## 5. Conclusions

In this work, we assessed the combined effect of size and surface charge on the interaction of nanoparticulate matter with mucus-mimicking mucin hydrogels. Non-PEGylated 30 nm particles exhibited greater diffusion in less crosslinked hydrogels that mimic healthy mucus when compared to the more densely crosslinked. Conversely, this effect was not observed by the 100 nm counterparts. PEGylated nanoparticles exhibited enhanced diffusion compared to citrate-stabilized particles, especially within the first 5 min of the assay. Among PEGylated formulations, neutral and negatively charged 30 nm nanoparticles generally showed greater diffusion than their positively charged counterparts in hydrogels mimicking mucus, highlighting the role of electrostatic and steric interactions with the mucin network. In contrast, for the 100 nm PEGylated particles, the neutral form showed higher diffusion than the positively charged one. These findings provide valuable insights into the design of surface-functionalized nanocarriers for efficient drug delivery across mucosal barriers and underscore the importance of tuning both nanoparticle size and surface charge, while considering the intrinsic properties of the mucus according to the body site and medical condition.

## Figures and Tables

**Table 1:** Characterization of pristine and PEG surface-modified 30 and 100 nm gold nanoparticles, as determined by DLS at 25 °C.

Nanoparticle	Surface Modification	Z-Average ± S.D. (nm)	D*_h_* by Intensity ± S.D. (nm)	D*_h_* Size by Number ± S.D. (nm)	PDI ± S.D.	Z-Potential ± S.D. (mV)
30 nm	Citrate	26 ± 6	27 ± 5	12 ± 1	0.15 ± 0.05	−18 ± 3
	PEG	36 ± 3	42 ± 5	20 ± 4	0.24 ± 0.01	−3 ± 1
	PEG-CM	34 ± 4	40 ± 1	18 ± 3	0.22 ± 0.01	−20 ± 2
	PEG-NH	43 ± 5	54 ±5	19 ± 2	0.32 ±0.02	+9 ± 1
100 nm	Citrate	96 ± 5	120 ± 2	63 ± 1	0.21 ± 0.01	−17 ± 1
	PEG	113 ± 2	110 ± 4	77 ± 2	0.20 ± 0.03	−4 ± 1
	PEG-CM	111 ± 4	108 ± 2	70 ± 2	0.19 ± 0.01	−16 ± 1
	PEG-NH	120 ± 5	129 ± 3	73 ± 2	0.18 ± 0.04	+17 ± 1

D*_h_*: hydrodynamic diameter; PDI: polydispersity index; Z-potential: zeta-potential.

## Data Availability

The original contributions presented in this study are included in the article/supplementary material. Further inquiries can be directed to the corresponding author.

## Supplementary Materials

The following supporting information can be downloaded at https://www.mdpi.com/article/10.3390/ph18101498/s1. Figure S1: Size distribution by intensity of 100-nm nanoparticles, as determined by NTA; Video S1.
