# Supplementary material for: Combined Effect of Size and Charge on the Interaction of Nanoparticles with Mucus-Mimicking Mucin Hydrogels
Source: Pharmaceuticals (Basel). 2025 Oct 5;18(10):1498. doi: 10.3390/ph18101498 (PMC12567317; doi:10.3390/ph18101498)
Supplement: Supplementary file 1 [file pharmaceuticals-18-01498-s001.zip › Porfiryeva et al-Supplementary information-Sept 10 2025.pdf]

Supplementary information

Combined Effect of Size and Charge on the Interaction of Nanoparticles with Mucus-Mimicking Mucin Hydrogels

Natalia N. Porfiryeva, Ivan Zlotver, and Alejandro Sosnik

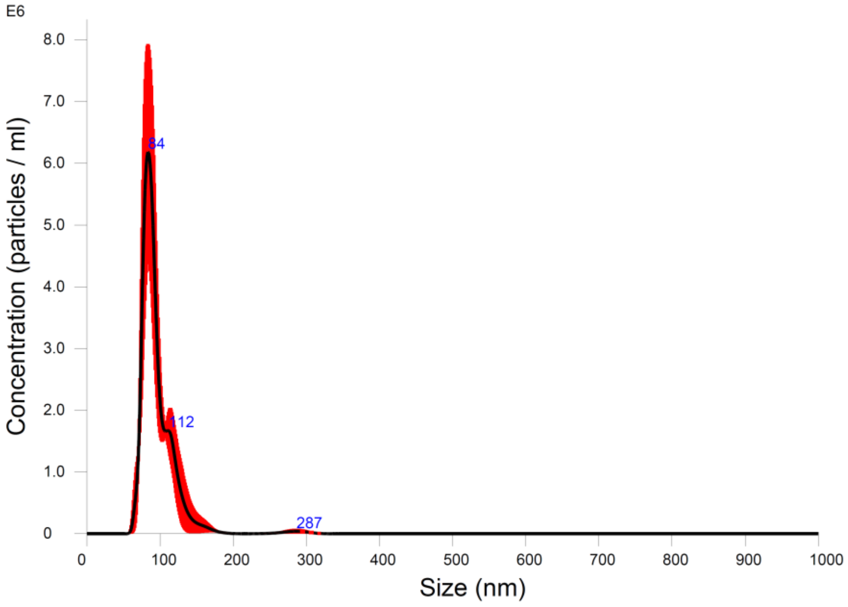

Figure S1. Size distribution by intensity of 100-nm nanoparticles, as determined by NTA.
